# PROMOTING AND LEVERAGING THE FULL POTENTIAL OF AN AGE-INCLUSIVE WORKFORCE

**DOI:** 10.1093/geroni/igae098.0409

**Published:** 2024-12-31

**Authors:** Katy Terveer

**Affiliations:** Age Wave, Orinda, California, United States

## Abstract

This session will focus on inspiring and informing the audience about how we can reimagine the way we work in order to benefit from the experience, skills, and wisdom of older workers. This session will elaborate on the Harvard Business Review article, “Redesigning Retirement: It’s time for a new deal between employers and older workers” written by the speaker (Katy Terveer) and her co-authors, Ken Dychtwald, PhD, and Bob Morison. We will explore the modern trends that are enabling longer workspans (i.e. the average number of years people spend in the workforce), such as the shift towards knowledge-based and service-based work and greater work flexibility. We will also explore the history of work and aging with an eye towards how ageism is holding us back in today’s workforce and what ageist myths we need to challenge. We will help aging advocates to understand and promote the benefits of increasing our workspans for individuals, employer organizations, and society. Finally, we will look at five key strategies that organizations can leverage to recruit and retain older workers as well as leverage the full potential of an age-inclusive workforce.

